# Links of Sleep Duration with Biomarkers of Accelerated Aging: the Baltimore Longitudinal Study of Aging

**DOI:** 10.1093/geroni/igab046.2512

**Published:** 2021-12-17

**Authors:** Emily Smail, Brion Maher, Ann Moore, Pei-Lun Kuo, Mark Wu, Dominique Low, Katie Stone, Adam Spira

**Affiliations:** 1 University of Florida, Baltimore, Maryland, United States; 2 Johns Hopkins Bloomberg School of Public Health, Baltimore, Maryland, United States; 3 National Institute on Aging, Baltimore, Maryland, United States; 5 Johns Hopkins University School of Medicine, Baltimore, Maryland, United States; 6 Stanford University School of Medicine, Stanford, California, United States; 7 University of California, Berkley, San Francisco, California, United States

## Abstract

Sleep disorders and sleep deprivation have been linked to markers of biological aging, including methylation change and increases in white blood cell and neutrophil counts. However, little is known regarding the association of sleep duration with biological markers of aging. We investigated links of self-reported sleep duration with biological aging markers in 615 participants in the Baltimore Longitudinal Study of Aging (BLSA) aged ≥50 years (mean = 71.0 ± 11.2, 49.6% women, 68.8% white) with data on self-reported sleep duration in hours (i.e., ≤6 (n=131), >6 to 7 (n=234), >7 (n=250)), demographics, and genetic and methylation data (mDNA). Our aging biomarker outcomes were four epigenetic clocks (Horvath, Hannum, PhenoAge, and GrimAge), mDNA-estimated PAI1, and estimated granulocyte count. After adjustment for age, sex, and race, compared to those sleeping ≤6 hours, those reporting >7 hours of sleep had faster biological aging according to Hannum age-acceleration, PhenoAge, GrimAge, mDNA-estimated PAI1, and granulocyte count. In addition, sleep duration interacted with age, such that compared to individuals reporting ≤6 hours of sleep, individuals reporting >6 to 7 hours showed lower GrimAge with increasing age, and with sex, such that males with longer sleep duration (>6 to 7 and >7 hours) showed a lower granulocyte count compared to females. Findings suggest that both short and long sleep duration are associated with and may contribute to accelerated aging. Prospective studies in larger samples are needed to examine whether changes in sleep duration precede changes in aging biomarkers.

